# High-resolution NMR in the native state

**DOI:** 10.1107/S2052252517002846

**Published:** 2017-02-23

**Authors:** Marc Baldus

**Affiliations:** a NMR Spectroscopy, Bijvoet Center for Biomolecular Research, Utrecht University, 3584 CH Utrecht, The Netherlands

**Keywords:** in-cell NMR, nuclear magnetic resonance, cellular structural biology, cellular environment, protein interactions

## Abstract

High-resolution NMR provides increasing possibilities to probe biomolecular structure in the native state. In this issue, Luchinat and Banci [*IUCrJ* (2017), **4**, 108–118] discuss progress on using in-cell NMR in prokaryotic and eukaryotic cell preparations.

One of the unique properties of magnetic resonance (MR) has been its ability to non-invasively yield molecular information from the test tube to applications in cells, tissue and even entire organisms. In the context of MR imaging (MRI) or spectroscopy (MRS), water or other small molecules including metabolites can be used as MR-active probes to infer localized chemical information at the micrometre scale.

In parallel, nuclear magnetic resonance (NMR) spectroscopy has become a well established structural biology approach to comprehensively study (at the atomic level) three-dimensional molecular structure and dynamics under *in vitro* solution-state or, more recently, solid-state NMR conditions. Applying the full and growing arsenal of such NMR methodology for studying larger molecules in a native (biological) setting has, however, required the development of novel and tailored NMR-based cellular structural biology concepts. Compared to the *in vitro* case, NMR studies under native conditions have to simultaneously address several challenges. Firstly, adequate spectroscopic sensitivity is of utmost importance as the native cellular environment is usually far more complex than what can be mimicked under well defined test-tube conditions. Another challenge relates to the fact that NMR signals of interest must be detected in a complex, usually dominating, molecular background. In practice, concepts suppressing signals of the surrounding molecular background so far have employed a combination of tailored biochemical, cell biology and spectroscopic methods. Thirdly, studying ‘live’ cell preparations furthermore calls for NMR approaches that allow NMR data to be rapidly acquired or involve instrumental setups that keep cells viable by allowing exchange of nutrients and removal of metabolic byproducts.

In their review in this issue of **IUCrJ**, E. Luchinat and L. Banci (Luchinat & Banci, 2017 ▸) give a timely and comprehensive overview of the field of in-cell NMR that allows for the study of molecular structure and dynamics in a cellular environment. In particular, they review progress in studying proteins and DNA in living cells. While initial work focused on proteins expressed in bacterial cells, protocols have been developed in recent years to study proteins or nucleic acids inside *Xenopus lavis* oocytes (using microinjection) and in human cells. In the latter case, Luchinat and Banci discuss various protein delivery methods that allow proteins or other biomolecules to be incorporated into cells as well as intracellular protein expression approaches. With these developments, solution-state NMR has become a powerful tool for studying three-dimensional molecular structure and the effect of molecular crowding at the atomic level. In addition, their review highlights elegant studies probing post-translational modifications and (mis)folding of proteins in living cells.

An inherent requirement for such solution-state NMR studies refers to the fact that the intrinsic molecular tumbling of the biomolecule to be studied is sufficiently fast. In cases where larger molecular units are to be studied, solid-state NMR provides increasing opportunities for studying complex molecular systems under (near) native conditions. Again, promising applications have been seen in recent years. Next to the work discussed by Luchinat and Banci, such studies have targeted large protein complexes embedded in bacterial cell envelopes (Kaplan *et al.*, 2015 ▸; Ward *et al.*, 2015 ▸) or provided structural insight into intact biomaterials (Chow *et al.*, 2014 ▸; Jantschke *et al.*, 2015 ▸). In fact, the advent of novel three-dimensional cell-culture methods provides further exciting opportunities for in-depth studies of molecular structures in a tissue setting, such as that elegantly shown by Duer *et al.* (Chow *et al.*, 2014 ▸) using various tissue preparations. Obviously, such studies can be readily combined with other cellular imaging methods such as ultra-high resolution light microscopy and electron tomography.

Finally, hybrid approaches that bridge the gap between *in vitro* and *in vivo* NMR can be of great value in dissecting molecular structure and interactions in the native state. For example, successful demonstrations included the use of cell lysates (Frederick *et al.*, 2015 ▸; Smith *et al.*, 2015 ▸) as well as *in vivo* extracts (Qiang *et al.*, 2017 ▸). Again, such studies can be readily combined with other biophysical methods including size-exclusion chromatography, hydrogen–deuterium exchange and electron paramagnetic resonance as well as with various cell biology techniques. Clearly, additional methodological progress in NMR will be needed to address current limitations such as protein concentration levels and cell viability aspects. However, the progress described by Luchinat and Banci suggests that high-resolution NMR in the native state is likely to provide an important and highly flexible tool in the emerging field of cellular structural biology in the future.
